# Negative affect mediates the association between negative news exposure and eye-tracking measures of attentional bias to food and alcohol cues among college students

**DOI:** 10.1371/journal.pone.0342572

**Published:** 2026-04-30

**Authors:** John Brand, Catherine Stanger, Caroline Borowy, Elizabeth Shelto, Delaina Carlson, Min-Jeong Yang, Diane Gilbert-Diamond

**Affiliations:** 1 Department of Epidemiology, Geisel School of Medicine at Dartmouth College, Hanover, New Hampshire, United States of America; 2 Center for Technology and Behavioral Health, Geisel School of Medicine at Dartmouth College, Hanover, New Hampshire, United States of America; 3 Department of Biomedical Data Science, Geisel School of Medicine at Dartmouth College, Hanover, New Hampshire, United States of America; 4 Rutgers Institute for Nicotine & Tobacco Studies, Health Behavior, Society & Policy, Rutgers School of Public Health, New Brunswick, New Jersey, United States of America; 5 Department of Medicine, Geisel School of Medicine at Dartmouth College, Hanover, New Hampshire, United States of America; 6 Department of Pediatrics, Geisel School of Medicine at Dartmouth College, Hanover, New Hampshire, United States of America; Methodist University Cape Fear Valley Health School of Medicine, UNITED STATES OF AMERICA

## Abstract

**Objective:**

To test the novel hypothesis that negative news exposure may lead to an increase in negative affect, which in turn leads to an increase in the amount of attention given to food and alcohol cues.

**Method:**

Forty-two college students made two laboratory visits, approximately 1-week apart. During each visit, students watched 15 minutes of short news clips while having their eye movements monitored. Participants were randomized to view either negative or control news during their first visit and watched the other news condition during their second visit. Food, alcohol, and control commercials were randomly shown between each news clip. At the end of each commercial, a static image of the branded food, alcohol, or control product appeared in middle of the screen and stayed visible for 5 seconds. We recorded two robust measures of attentional bias to the static image: first fixation bias and cumulative fixation bias. The Maastricht Momentary Mood Questionnaire (3MQ) was completed prior to the news clips being shown to record a baseline level of negative affect. Participants completed the 3MQ again following viewing the news clips to measure changes in negative affect.

**Results:**

In a series of linear regressions, we found that, overall, viewing negative news was associated with a statistically significant increase in negative affect (β = 0.72, P < 0.001), as well as a statistically significant increase in cumulative fixation bias (P = 0.041) and a nonsignificant increase in first fixation bias (P = 0.074) to food and alcohol vs. control cues. Furthermore, our data suggests that negative affect may partially mediate the association between negative news exposure and cumulative fixation bias, though the mediation effect did not reach statistical significance (β = 0.14; P = 0.056).

**Conclusion:**

In conclusion, exposure to negative news media increased negative affect and attentional bias to food and alcohol cues among college students, suggesting that distressing media may contribute to maladaptive coping behaviors such as emotional eating and alcohol use.

## Introduction

One in three college-aged Americans have obesity [1], with higher rates in later years of college [2,3], which increases the risk for adiposity-associated chronic disease and all-cause mortality [4]. College is a transitional time for young adults, often accompanied by unhealthy changes in physical activity, diet and other energy balance related behaviors. Problematic drinking behaviors, defined as excess consumption (i.e., seven drinks per week or more for women and 14 or more for men) or binge drinking (i.e., more than three drinks per occasion for women and more than four for men) are also common in college students, with almost 33 percent of college students reported engaging in problematic drinking within the past 30 days at the time of being surveyed [5]. Problematic drinking during college is linked to injury and accidental death, unintended pregnancy, and future alcohol abuse [6–8]. and may contribute to the transition from healthy weight to overweight/obesity [9]. Understanding and preventing obesogenic behaviors and problematic drinking among college students may help them to develop healthy habits that could positively affect their lifelong health.

Both food and alcohol consumption are promoted by the dopaminergic mesolimbic reward system in the brain that detects rewarding stimuli and prompts cravings and reward-seeking behaviors [10–13]. This pathway is involved in classical conditioning [14], a learning process where paired exposure to a cue and an associated reward can subsequently lead to motivation for that reward upon cue exposure. Both food and alcohol function as primary reinforcers in operant conditioning [15–18], and exposure to images of food and alcohol activate the brain reward pathway and may result in the development of an attentional bias to food and alcohol cues in in the environment [19,20].

While research supports the importance of attentional bias in the etiology of obesity and problematic drinking behaviors [21–23],^,^ there is little research investigating risk factors for increased cue reactivity among college students, despite their heightened risk for obesity and problematic drinking relative to their same age non-college peers [24–26]. We propose a model in which ubiquitous negative news exposure may lead to an increase in negative affect, which in turn, leads to an increase in the amount of attention given to food and alcohol cues to alleviate the increase in negative affect. Our model is grounded in incentive salience theory that describes how rewards and their cues become highly motivating and attention grabbing through associate learning principles [27]. Negative affect activates neural systems involved in both threat detection and reward processing, particularly the amygdala and ventral striatum [13,28,29]. This emotional arousal increases attentional salience of emotionally relevant or rewarding cues (such as food or alcohol), leading to a heightened attentional bias [21,22,30]. Attentional bias toward these cues may function as a maladaptive emotion regulation strategy, offering temporary relief or distraction from distress [27,31]. Over time, this pattern can reinforce maladaptive coping strategies, particularly in environments that are replete with food and alcohol cues [30,32].

Negative news exposure is related to decreased positive affect and increased negative affect in observational research [33–36]. While compelling, the observational nature of these previous studies, preclude inference on the temporality of associations and causation. Specifically, it is unknown whether negative news exposure caused a change in negative affect and/or if those with a negative affect increased their negative news exposure in response. The specific effect of news from social media, as opposed to more traditional news sources, on affect is also unknown. Social media is a frequent news source for many college students; a 2018 survey of 5, 844 students from 11 U.S. institutions reported that 89% viewed news from social media at least once a week and 72% viewed news on social media daily [37]. News on social media tends to be overwhelmingly negative and sensationalized to target people’s emotions [38–47].

Recognizing college students ubiquitous use of social media, food marketers are increasingly advertising via social news feeds [42,43], with approximately 68% of advertisements shown in the United States promoting unhealthy foods and sugary drinks [44]. This high exposure may lead to a further sensitizing of an individual to food and alcohol advertisements, increasing their attentional bias to food and alcohol cues leading to increased craving and subsequent reward seeking and consumption behavior. The saliency of the cues may also be heightened under cognitive strain such as negative mood, leading to an increased attentional bias [45]. In this study, we explore the novel hypothesis that negative news exposure leads to an increase in attentional bias to food and alcohol cues among college students through the mechanism of negative affect. We measure two robust eye tracking measures of attentional bias related to unconscious and conscious processing. Unconscious attentional bias is automatic and reflects how much a salient cue (e.g., food or alcohol stimuli) captures attention and is measured as first fixation bias [46]. Conscious attentional bias is under conscious control and reflects the maintenance of attention to a salient cues and is measured as cumulative fixation bias [46,47]. We hypothesize that negative affect will increase following acute negative news exposure, and further, that attentional bias to food cues will be greater when participants are exposed to negative vs. control news media. We also hypothesized that negative affect will partially mediate the association between negative news exposure and attentional bias to food cues; the association between negative news and attentional bias to food cues will be strongest for those with the greatest levels of negative affect.

## Methods

### Study design

Forty-two undergraduate college students aged 18–27 years were recruited through flyers posted on-campus. There were no exclusion requirements. Participants completed 2 laboratory visits approximately 1 week apart between June 1, 2023 and February 27, 2024. During each visit, students watched 15 minutes of short news clips on an Apple iPhone while their eye movements were simultaneously monitored. This study was a within-subjects crossover design. Within-subjects designs are often preferred in psychological research because they control for individual differences and increase statistical power [48,49]. Participants were randomized to view either negative or control news during their first visit and received the other condition during their second visit. Three 30-second commercials for branded food, alcohol or control (e.g., toiletry, laundry detergent) products were embedded within the news clips at random. At the end of each commercial, a static image of the advertised item appeared in middle of the screen and stayed visible for 5 seconds. We recorded two robust measures of attentional bias to the static image: first fixation bias and cumulative fixation bias [46]. Eye movements were collected using SR Research’s weblink system (SR Research, Mississauga, ON, Canada), which allows for natural user interaction on mobile devices via a framing device that holds the phone. The Maastricht Momentary Mood Questionnaire (3MQ) [50,51] was completed prior to the news clips being shown to record baseline level of negative affect. Participants completed the 3MQ again following the clips to measure changes in negative affect. All study procedures were approved by The Committee for the Protection of Human Subjects at Dartmouth College (#32702; original approval April 2023). Participants provided written consent.

### Negative news categories

Negative news stories contained contemporary news at the time of data collection and included: climate change, democracy threat, war in Ukraine, politics, mass shootings, terrorism and Israel, abortion, floods and wildfires, Covid-19/long covid, college debt, the job market, and inflation, recession, and debt ceiling. Participants were shown one clip from each category for a total of 14 negative news clips with an exposure time of 15 minutes and 24 seconds.

### Neutral news categories

Most news stories exist on a continuum between positive and negative, with little or no news being truly neutral. Therefore, we used previously published criteria [52] to select control news clips from a list of neutral to positive categories, including current stories about natural phenomena, community heroes, pets, charity work, and good Samaritans. Participants were shown a total of 14 news clips with an exposure time of 15 minutes and 19 seconds.

### Food and alcohol commercials

The food commercials shown to participants were Oreo and Mountain Dew. The alcohol commercials shown were Bud light and Wiser’s whiskey. All commercials were matched for duration.

### Attentional bias to food and alcohol cues

To assess attentional bias to food cues, we calculated first fixation bias by subtracting the average amount of time of the first fixation on a branded food image from the average amount of time of the first fixation on a control branded image. We also calculated cumulative gaze duration bias as the average dwell time of all fixations on food branded images minus the average dwell time of all fixations on control cues. Changes in attentional bias metrics were computed for control and negative news conditions, by subtracting metrics in the negative condition from those in the control condition [53,54]. A positive number indicates higher attentional bias toward food following negative news exposure compared to control news exposure. This procedure was repeated using alcohol cues to calculate first fixation bias and cumulative fixation bias to alcohol cues for each condition, respectively.

### Negative affect

The Maastricht Momentary Mood Questionnaire (3MQ) was used to measure negative affect [51]. The questionnaire consists of six negative emotions (insecure, lonely, anxious, irritated, down, and guilty). Respondents indicated to what extent they are currently experiencing these emotions on a 7‐point Likert scale (1 = not at all; 7 = very). This scale has good reliability, Cronbach’s alpha = 0.8 [51]. Affect scores were computed for pre and post news exposure in each condition. We also computed change in negative affect scores (ΔAffect) for control and negative news conditions, by subtracting the pre score from the post score. A positive number indicates an increase in negative affect following news exposure.

### Other measures/covariates

Students reported on their sex, age, and race/ethnicity. Participants also completed the Inside-Out-Screener (IOS), a 6-item digital screening tool designed to assess broad eating disorder risk and symptomatology, validated for individuals aged 14 and over [55]. It is not a diagnostic tool but can be used to indicate risk for eating disorders.

A score at or above 19 indicates at risk for an eating disorder. Participants also completed the alcohol use disorders identification test (AUDIT) to screen for alcohol abuse risk [56]. Like IOS, it is not diagnostic but can indicate hazardous consumption risk. A score of 15 or above indicates increased hazardous alcohol consumption risk.

### Statistical analysis

We conducted all analyses using the R language and environment for statistical computing. All analyses were computed using first fixation bias, cumulative fixation bias, and post negative affect as the outcomes, and news condition as the exposure.

Distributions of first fixation bias and cumulative fixation bias were compared across age, sex, and race/ethnicity. We also evaluated the association between IOS scores and attentional bias to food cues, and the association between the AUDIT and attentional bias to alcohol cues. All adjusted analyses included age and sex because they have been associated with increased attentional bias to food cues in other studies [21,22]. The association between first fixation bias and cumulative fixation bias was assessed by computing Pearson’s correlation coefficient using the *stat* package*.* Because our outcomes were collected using different scales, first fixation bias, cumulative fixation bias, and post negative affect were standardized across all trials using the *scale* function prior to all analyses. Prior to conducting our main analyses, an outlier analysis was conducted to identify change in negative affect scores and attentional bias scores that were ±3.0 standard deviations from the mean. There were 5 statistical outliers related to negative affect scores. To evaluate if these outliers had undue influence on our results, we repeated all analyses with the outliers removed.

We plotted the change in affect after exposure to negative news and control news conditions using a box and whiskers plot. We then compared mean change in affect between negative and control news conditions using a dependent samples t-test. To assess if first fixation bias increased following news exposure, a linear mixed effect regression was computed to predict first fixation bias from news condition coded as binary (0 = control; 1 = negative). Stimuli cue (0 = Food; 1 = Alcohol) was entered as binary fixed effect and participant as a random effect. Separate linear mixed effects regressions were then computed to assess the direct effect between i) negative affect and negative news exposure, and ii) negative affect with attentional bias. The association between attentional bias and negative affect was assessed by predicting first fixation bias or cumulative fixation bias from post negative affect controlling for pre negative affect. Condition (0 = control; 1 = negative) and cue type (0 = Food; 1 = Alcohol) were coded as binary fixed effects and participant as a random effect. To assess if negative news exposure was associated with negative affect, post negative affect was predicted by condition coded as binary, controlling for pre negative affect and participant as a random effect. We then calculated the standardized indirect effect between negative news exposure and first fixation bias through post negative affect (controlling for prior negative affect) using the bootstrapping analysis in the *mediate* package. The number of bootstrapped samples was set to 500, and the set.seed(1) function was used to make the bootstrapping sample reproducible. All analyses were then repeated using cumulative fixation bias as the outcome. As a sensitivity analysis, we also computed these analyses for food and alcohol cues separately.

## Results

Most participants were male (57.14%), white, non-Hispanic (88%), and the average participant age was 20.48 (SD = 1.80; range = 18–26). Participant age, sex, and ethnicity were not statistically associated with either first fixation bias or cumulative fixation bias to food or alcohol cues. The mean of the IOS screener was 9.28 (SD = 8.22) and the mean of the AUDIT was 3.23 (SD = 4.64). None of our participants had scores to indicate increased eating disorder or hazardous alcohol consumption risk. There were no associations between IOS screener scores and attentional bias to food cues, nor any associations between AUDIT scores and attentional bias to alcohol cues. First fixation bias and cumulative fixation bias were positively correlated (P < 0.001) (Table 1).

**Table 1:** Bivariate associations of attentional bias with age, sex, race, and problematic food and alcohol screeners.

		Attentional bias to food and alcohol cues	
		First fixation bias (msec)	Cumulative fixation bias (msec)
	N	Mean (SD)	Mean (SD)
Overall	42		
Age (years)^1^			
18-19	12	−28.53 (335.60)	−39.71 (493.24
20	2	89.10 (327.67)	237.18 (547.95
21	14	56.11 (242.40)	−97.5 (373.95)
>22	9	204.31 (217.93)	311.52 (464.04)
		0.347	
Sex^2^			
Male	18	85.41 (304.25)	170.5 (483.83)
Female	24	53.01 (278.39)	−25.86 (482.94)
		*0.722*	*0.201*
Race^3^			
White, non-Hispanic	37	67.41 (305.05)	370.51 (281.81)
Other, non-Hispanic	5	61.05 (179.44)	224.46 (242.94)
		*--*	*--*
IOS Screener^4^	42	*P* = 0.651	*P* = 0.934
AUDIT^5^	42	P = 0.129	P = 0.271

^1^P-value calculated to test for linear trend. Age range = 18–27.

^2^P-value calculated using independent samples t-test

^3^P-values not calculated because of sample size differences. Descriptives shown for transparency.

^4^P-value calculated from linear regression predicting first fixation bias or cumulative fixation bias to food cues from inside our screener (IOS).

^5^P-value calculated from linear regression predicting first fixation bias or cumulative fixation bias to alcohol cues from AUDIT scores.

**Fig 1:**
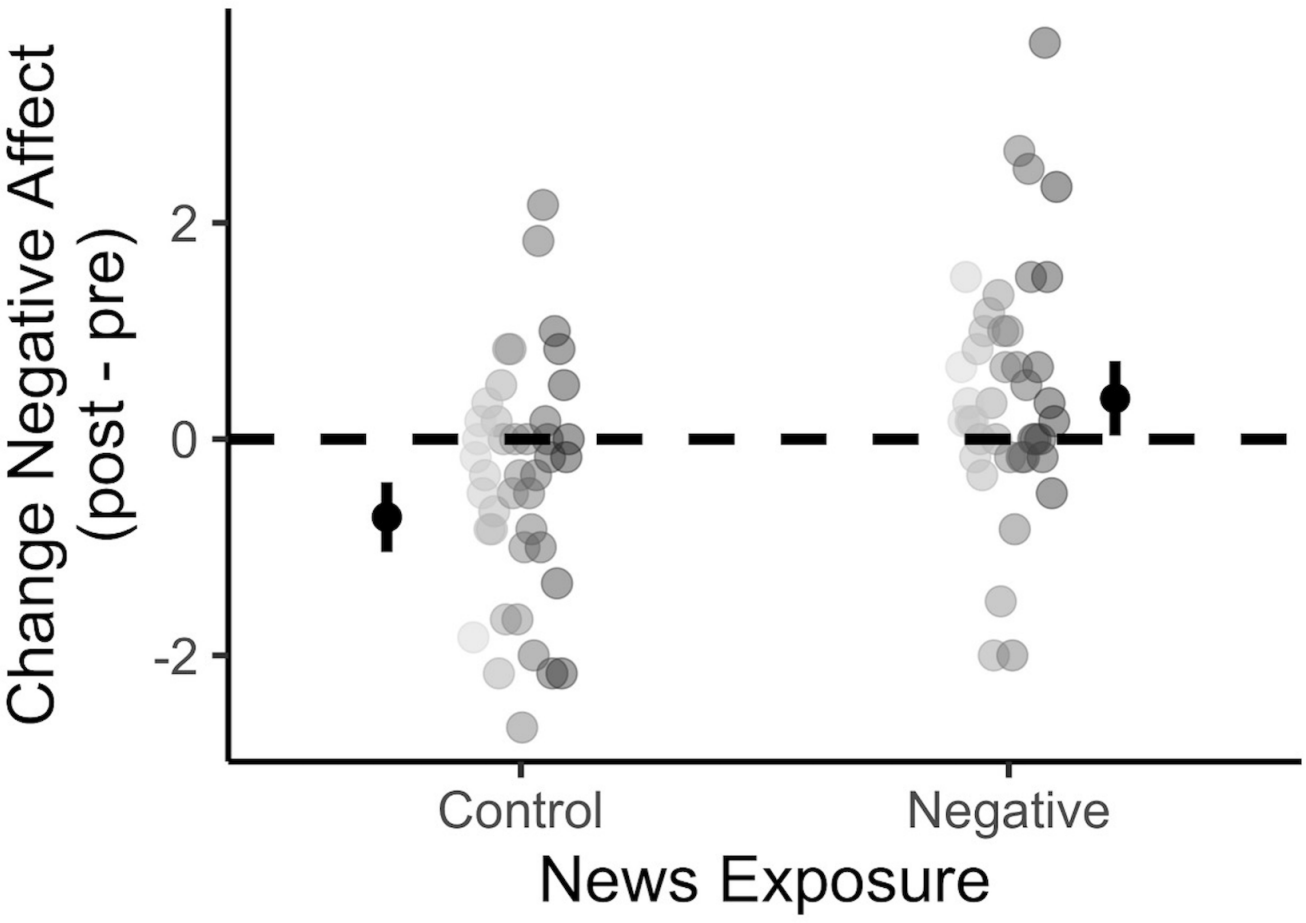
Post – pre negative affect change by news exposure condition. Circles represent individual data points. Error bars represent the 95% CI around mean estimates.

**Fig 2:**
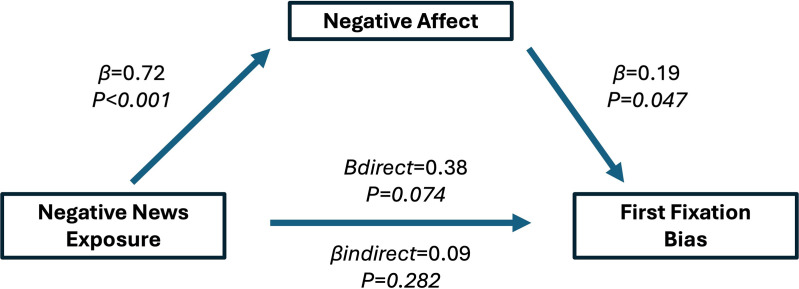
Standardized regression coefficients for the relationship between cumulative fixation bias and negative news exposure as mediated by negative affect. Adjusted for age and sex.

Overall, viewing negative news was associated with statistically significant increase in negative affect compared to viewing control news (P < 0.001; Cohen’s d = 0.57) (Fig 1). In a series of sex and age adjusted mixed effect regressions using cumulative fixation bias as the outcome, the standardized regression coefficient between negative news and cumulative fixation bias was statistically significant, as was the regression coefficient between negative affect and cumulative fixation bias. Viewing negative news was associated with a statistically significant 0.25 SD (P = 0.041) increase in cumulative fixation bias, and for every 1 SD increase in negative affect there was a statistically significant 0.21 SD (P = 0.049) increase in cumulative fixation bias. Furthermore, the standardized indirect effect of negative affect between negative news exposure and cumulative fixation bias was not significant (β = 0.14 SD; P = 0.056) (Figs 1, 2).

**Fig 3:**
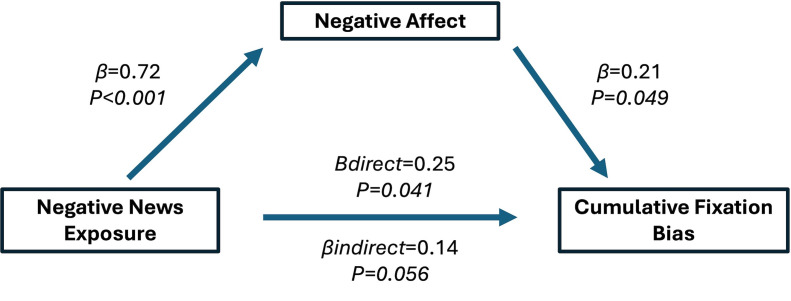
Standardized regression coefficients for the relationship between first fixation bias and negative news exposure as mediated by negative affect. Adjusted for age and sex.

In a series of sex and age adjusted mixed effect regressions using first fixation bias as the outcome, viewing negative news was associated with a non-statistically significant 0.38 SD increase in first fixation bias (P = 0.074). Negative affect was statistically significantly associated with first fixation bias; for every 1 SD increase in negative affect there was a 0.19 SD (P = 0.047) increase in first fixation bias. However, the standardized indirect effect of negative affect between negative news exposure and first fixation bias was not significant (β = 0.09; P = 0.282) (Fig 3).

The findings were not substantively changed, and the statistical significance of all analyses remained unchanged after outlier removal. The findings were not substantively changed when analyses were completed separately for food and alcohol cues, although the results did not remain statistically significant, more than likely due to a lack of power.

## Discussion

Among a sample of college students, we investigated the direct and indirect effects of negative affect between negative news exposure and eye tracking measures of attentional bias. We found that direct exposure to acute negative news was associated with a statistically significant increase in negative affect. However, there were no statistically significant associations between negative news and first fixation bias and cumulative fixation bias to combined food and alcohol cues, and there was not a statistically significant mediation effect of negative affect on the associations between negative news exposure and first fixation bias and cumulative fixation bias.

While associations between negative news and attentional bias measures were non-significant and should be interpreted cautiously, the trends suggest possible underlying associations. Given the exploratory nature of the study and the potential for small effect sizes in attentional bias paradigms [47,53,54], the lack of significance may reflect insufficient sample size rather than the absence of a true effect. For example, these near significant associations are consistent with work showing that inducing a negative mood may increase selective attention to food cue [45], as well as increase in attentional bias for alcohol-related cues [23].

Additionality, our finding of significant associations between negative affect and attentional bias is consistent with research suggesting that this association may play an important role in maladaptive eating and drinking behaviors. Coping with negative affect is a key component of etiological models of maladaptive alcohol and food consumption. These models posit that individuals experiencing high levels of negative affect eat or consume alcohol to relieve the stress experienced from negative emotions [57–59]. This may be attributed to a negative reinforcement mechanism in which the negative affect is reduced in the short term. However, it is unclear whether this is an effective long-term strategy to reduce negative affect and may even result in an overall increase in negative affect over time. For example, framing information in the negative captures attention [60] and college students who learn about adverse breaking news via social media are more likely to experience cognitive preoccupation with breaking news, which may lead to more compulsive social media use [61]. It is therefore possible that negative news’ availability in the modern media environment leads to increased engagement in social media among college students. Future research is needed to evaluate the effect of long-term news exposure on both acute and chronic negative affect.

First fixation bias and cumulative fixation bias are hypothesized to measure distinct aspects of early and late attentional mechanisms, respectively. Contrasting past work, we found a strong correlation between the two, suggesting a common underlying attentional mechanism. However, it is possible that this association is an artifact of the way in which we measured attention to our study stimuli. We calculated attentional bias by contrasting eye tracking measures to experimental stimuli with those to control stimuli that were shown in isolation. This contrasts typical eye tracking paradigms of attentional bias that either use cueing paradigms or multiple stimuli displays that increase the demands on attention. First fixation bias measures how much a stimulus initially captures attention, whereas cumulative fixation bias how much a stimulus holds attention over time. The lack of competing stimulus in our study may have biased how our participants allocated their attention to target stimuli both initially and over time, and may reflect general attentional engagement with salient stimuli rather than a true bias in attentional competition.

While this may limit construct validity—particularly in terms of measuring attention under competition – it was an intentional design choice to enhance ecological validity. In everyday settings such as scrolling through social media or browsing news content, individuals often engage with single, sequential stimuli rather than competing images presented simultaneously. By mimicking this more naturalistic viewing experience, our design provides insight into how attention is allocated to emotionally salient cues in real-world media environments. This trade-off highlights the importance of matching task design to research goals—whether to isolate attentional processes or to approximate real-world exposure—and suggests future studies should consider incorporating both isolated and competing stimulus paradigms to triangulate attentional bias effects.

These findings have important practical implications for health promotion and intervention design, particularly among young adults. Given that negative news exposure can heighten emotional distress and drive attention toward unhealthy reward cues, strategies that reduce exposure to distressing media or buffer its emotional impact may help mitigate maladaptive behaviors such as emotional eating and alcohol use [62,63]. Interventions such as mindfulness, cognitive reappraisal, or stress management training may reduce the impact of negative affect on attentional processes and subsequent behavior [64–66]. Media literacy programs that train individuals to critically evaluate media content and understand its emotional effect may serve as effective interventions to reduce vulnerability to maladaptive coping [67,68], particularly in high-exposure environments such as college campuses.

Our results should be understood within the following limitations. We did not assess repeated news exposure; despite the fact it may be an additional risk factor to increased attentional bias. Relatedly, the structure of our experiment precludes conclusions about the dose response effects of negative news exposure. Future research is needed to measure changes in negative affect throughout negative news exposure, possibly using biometrics. We also didn’t collect data on participants’ alcohol or food use as coping strategies or measure actual consumption. This limits our ability to interpret attentional biases in negative affect as real-world emotion regulation strategies or consumption patterns. Future studies should include these measures to understand attentional bias’s functional relevance in coping behaviors and whether attentional shifts lead to increased consumption under emotional distress. We also had a majority white, non-Hispanic population. Epidemiological studies show problematic drinking volume varies by U.S. ethnic group, with rates highest among Hispanics and Native Americans followed by Whites, Blacks, and Asians [69]. Similarly, obesity disproportionately affects certain racial and ethnic minority groups, with Non-Hispanic Blacks at the highest risk [70]. Future work is needed to understand how our proposed relationships in high-risk groups. A further limitation is the restricted range of AUDIT and IOS screener scores in our sample, as no participants reported elevated problematic drinking or eating behavior. This limits generalizability. Future research should examine if associations between negative affect and attentional bias are stronger in individuals with clinically relevant alcohol or food-related behavior. We also observed near significant associations between negative news exposure and attentional bias, suggesting possible underlying associations. Further investigation with a-priori power calculations is warranted to ensure adequate sample size and sensitivity to detect subtle effects.

## Conclusion

This study provides novel evidence linking negative news exposure to attentional bias toward food and alcohol cues, with negative affect playing a mediating role. These findings underscore the psychological and attentional consequences of distressing media and highlight potential pathways by which such exposure may contribute to maladaptive behaviors, particularly among college students. While our naturalistic design enhances ecological validity and offers insights into real-world attentional processes, future research should expand on these results by examining repeated exposure, consumption behaviors, and responses in more diverse populations. Addressing these gaps will be critical for informing targeted interventions—such as emotion regulation training and media literacy programs—that aim to buffer the cognitive and behavioral effects of negative media exposure in vulnerable groups.
